# A Chlorfenapyr Mixture Net Interceptor^®^ G2 Shows High Efficacy and Wash Durability against Resistant Mosquitoes in West Africa

**DOI:** 10.1371/journal.pone.0165925

**Published:** 2016-11-16

**Authors:** Raphael N’Guessan, Abibatou Odjo, Corine Ngufor, David Malone, Mark Rowland

**Affiliations:** 1 London School of Hygiene and Tropical Medicine, London, United Kingdom; 2 Centre de Recherche Entomologique de Cotonou, Ministry of Health, Cotonou, Benin; 3 Pan-African Malaria Vector Research Consortium (PAMVERC), London, United Kingdom; 4 Innovative Vector Control Consortium, Liverpool, United Kingdom; University of Crete, GREECE

## Abstract

**Background:**

Malaria control through use of long-lasting insecticidal nets (LN) is threatened by the selection of anopheline mosquitoes strongly resistant to pyrethroid insecticides. To sustain future effectiveness it is essential to identify and evaluate novel insecticides suitable for nets. Mixtures of two insecticides with contrasting mode of action have the potential to kill resistant vectors and restore transmission control provided the formulation can withstand regular washing over the net’s life span.

**Method:**

The efficacy of a novel mixture LN, Interceptor^®^ G2, that combines the pyrrole chlorfenapyr and pyrethroid alpha-cypermethrin was evaluated under controlled household conditions (experimental hut trial) and by laboratory bioassay against pyrethroid resistant *An*. *gambiae* in Benin before and after standardized washing. Comparison arms included standard alpha-cypermethrin LN, nets hand-treated with chlorfenapyr-only and untreated nets.

**Results:**

The chlorfenapyr-alphacypermethrin LN demonstrated improved efficacy and wash resistance compared to a standard alpha-cypermethrin LN against pyrethroid resistant mosquitoes (resistance ratio 207). In experimental hut trial alpha-cypermethrin LN killed only 20% (95% CI 15–26%) of host-seeking *An*. *gambiae* whilst mixture LN killed 71% (95% CI 65–77%). Mixture LN washed 20 times killed 65% (95% CI 58–71%), and thus intensive washing reduced efficacy by only 6% (95% CI 1.3–11%). The chlorfenapyr net killed 76% (95% CI 70–81%). Personal protection and blood feeding inhibition did not differ between mixture and pyrethroid LN; however, the mixture LN was 2.5 (95% CI: 2.1–3.1) times more protective than untreated nets. Standard WHO cone bioassays conducted during day time hours failed to anticipate field efficacy but overnight tunnel tests successfully predicted mixture LN and chlorfenapyr net efficacy in field trials.

**Conclusion:**

Interceptor^®^ G2 LN demonstrates the potential to control transmission and provide community protection over the normal lifespan of long lasting nets where standard pyrethroid LN show signs of failing due to resistance.

## Introduction

Long-lasting insecticidal nets (LN) are the most widely used public health interventions for malaria prevention [1]. In countries of sub-Saharan Africa the estimated proportion of people with access to LN in their homes in 2015 was 67% and the proportion sleeping under LN was 55% compared to less than 2% in 2000. Malaria infection prevalence among African children is estimated to have declined from 33% to 16% and under-5 all-cause mortality by 48% over the same period. The massive scale up of LN during the last decade has made a major contribution to these declines. Recent figures estimate that 427 million LN were distributed in Africa in 2012–2014. The current effectiveness of LN is entirely dependent on a single class of insecticide, the pyrethroids, and with the high level of LN coverage the selection pressure on mosquito vector populations is immense. Subsequently, resistance to pyrethroids has increased in distribution and strength [2, 3]. Of the 42 African countries deploying LN, pyrethroid resistance was reported in 29 countries in 2010–2015. While a negative epidemiological impact of pyrethroid resistance on malaria control has yet to be confirmed [1, 4], an increasing number of reports show that pyrethroid resistance is capable of reducing the effectiveness of LNs used against host seeking mosquitoes [5–7], and leading brands are giving less personal protection and reduced vector mortality rates compared to areas of pyrethroid susceptibility [8, 9].

If LN are to remain viable for malaria transmission control in the future it is essential that new public health insecticides are identified and developed to address the growing problem of resistance. One alternative insecticide to pyrethroids that is presently under development is chlorfenapyr, a member of the pyrrole class [10]. Unlike other adulticides chlorfenapyr is not neurotoxic but owes its toxicity to disruption of cellular respiration and oxidative phosphorylation in mitochondria [10]. Owing to its unique mode of action, chlorfenapyr is active against pyrethroid resistant and susceptible mosquitoes [11, 12]. On mosquito netting chlorfenapyr is toxic to mosquitoes but lacks the property of excito-repellency crucial for reducing mosquito biting rates and providing personal protection to net users [11]. On mosquito nets hand treated with the chlorfenapyr-pyrethroid mixture and tested in experimental huts the pyrethroid component provided excito-repellency whilst the chlorfenapyr component restored insecticidal activity against pyrethroid resistant mosquitoes [12, 13]. Such mixture nets have potential for insecticide resistance management because phenotypes that survive contact with one insecticide due to resistance should be killed by the other insecticide provided they are not resistant to both [14]. To date there is no recorded occurrence of resistance to chlorfenapyr in any species of anopheline or culicine mosquito [11–13, 15–18].

Based on the encouraging activity of the hand-treated mixture nets a product development partnership was established between BASF SE, the Innovative Vector Control Consortium and the London School of Hygiene & Tropical Medicine to develop a long-lasting mixture LN to combine chlorfenapyr and alpha-cypermethrin in a wash resistant formulation which would address the problem of malaria control in countries with high level insecticide resistance. The World Health Organization (WHO) sets criteria for LN to attain to be recommended for public health use. The WHO Pesticide Evaluation Scheme (WHOPES) stipulates that new candidate pyrethroid LN and mixture LN should achieve efficacy in experimental hut studies equal or better than standard LN subjected to 20 standardized washes before qualifying as fit for malaria control [19]. The WHO Vector Control Action Group (VCAG) which advises WHO on new vector control tools goes one step further and stipulates that for use against pyrethroid resistant vector populations the mixture LN should demonstrate efficacy (mosquito mortality or prevention of blood-feeding) significantly greater than standard pyrethroid-only LN [20].

The objectives of the studies reported here were to evaluate the efficacy of the mixture chlorfenapyr-alphacypermethrin LN, Interceptor^®^ G2 LN, in laboratory tests and experimental hut trial against a well characterized population of pyrethroid-resistant *Anopheles gambiae* according to the criteria of WHOPES and VCAG.

## Materials and Methods

### Study Site and Experimental Huts

The Phase II experimental hut station is situated in a rice irrigation zone near Cové (7°14′N, 2°18′E), Benin. The vector species are *Anopheles coluzzii* (formerly *An gambiae* M form) and *An*. *gambiae* s.s. (S form) [9]. The frequency of the 1014F *kdr* allele is 89% and the P450 CYP6P3 associated with metabolic resistance is also present.

The WHO Phase II evaluation of Interceptor G2 was performed in 8 experimental huts of the West African design [19]. The operating principle of the huts is to allow unrestricted access of host seeking mosquitoes though baffled entry slits and to capture exiting survivors in veranda traps, and to deny entry to scavenging ants.

### Insecticide resistance tests

Cylinder tests with WHO test papers were used to assess the frequency of pyrethroid resistance of mosquitoes, reared from sites near the huts [21]. To determine the intensity of resistance, the adults were tested in CDC bottle bioassays treated with a range of alpha-cypermethrin doses [22]. Mosquitoes were exposed for 1 h and mortality recorded 24 h later. *An*. *gambiae* s.l. were identified to species and genotyped for *kdr* resistance.

### Net treatments and trial procedure

The Interceptor^®^ G2 LN, Interceptor LN and chlorfenapyr formulation (Phantom 240g/L SC) were supplied by BASF SE. The target concentrations were 100 mg/m^2^ alpha-cypermethin and 200 mg/m^2^ chlorfenapyr on Interceptor^®^ G2 LN, 200 mg/m^2^ alpha-cypermethrin on Interceptor^®^ LN and 200 mg/m^2^ on the chlorfenapyr net. All nets were 100 denier polyester.

The following 8 treatment arms were compared in the experimental huts: Interceptor^®^ G2 LN unwashed, washed 15 times and washed 20 times; Interceptor^®^ LN unwashed, washed 15 times and washed 20 times; chlorfenapyr hand treated net; untreated net.

Washing of LNs was done according to WHO Phase II protocols [19]. The interval between washes was 1 day which is the established regeneration time for Interceptor G2 and Interceptor LN [23]. Each net was cut with six holes of 4 cm diameter to simulate wear and tear. Treatments were rotated between huts each week and sleepers rotated each night using a randomized latin square design to adjust for any variation in attractiveness to mosquitoes. Each morning mosquitoes were collected and held for a 72 h to record any delayed mortality.

The primary outcomes were: entry and exiting rates, percentage mortality relative to the total collected, percentage blood-feeding inhibition, percentage personal protection.

### Chemical analysis

Nets were sampled before and after washing and after the trial [19]. Determination of alpha-cypermethrin and chlorfenapyr content was performed at BASF SE applying CIPAC 454/LN method.

### Supporting bioassay tests on nets used in the trials

The efficacy of the 8 treatments was also tested under laboratory conditions applying standard WHO cone and tunnel bioassays against pyrethroid susceptible (Kisumu) and resistant (Cové) *An gambiae* strains.

For each type of net and wash point, 100 females were subjected to 3 min exposure in cone bioassays in replicates of 5 mosquitoes per cone and 150 were tested in tunnel tests in replicates of 50 mosquitoes per test in accordance with WHO guidelines [19]. The tunnels were divided into two sections by a netting frame punctured with 9 holes slotted across the tunnel. In one section an anaesthetized guinea pig was housed unconstrained in a cage to attract mosquitoes from the release section overnight. Test conditions were 27 ± 2°C and 75 ± 10% RH. Mosquito mortality was recorded after 24h and 72h holding periods.

### Statistical analysis

Estimates of LD50 and resistance ratios were generated using probit analysis (Polo Plus, LeOra Software). Cone and tunnel test were analyzed using logistic regression for grouped data adjusting for clustering within replicate tests (STATA/IC 12, Stata Corp., College Station, USA).

Proportional outcomes in the experimental hut trial (net penetration, blood-feeding, exiting and mortality) related to each treatment were assessed using binomial generalized linear mixed models (GLMM) with a logit link function fitted using the ‘lme4’ package for R (version 2.15.0). In addition to the fixed effect of each treatment, each model included random effects to account for variation between the huts, sleepers, the weeks of the trial, and for variation not explained by the other terms. Comparison between numeric outcomes (deterrence, personal protection) between treatments was analyzed using negative binomial regression with adjustment for variation in the covariates above.

### Ethical clearance

Approved by the Ethical Committees of LSHTM, UK and Ministry of Health, Benin. The experimental hut volunteer sleepers gave written informed consent before participating and were provided with chemoprophylaxis during the study

## Results

### WHO susceptibility tests and intensity dose–response bioassay

To determine the frequency and strength of resistance to pyrethroids in the local strain of *An*. *gambiae* s.l. colonized from rice fields near the experimental huts, adult mosquitoes 2–3 days old were tested in WHO cylinder tests and CDC intensity bottle bioassays [21, 22].

After confirming that exposure to diagnostic concentrations of pyrethroid insecticides would kill 100% of the susceptible *An gambiae* Kisumu strain, *An gambiae* s.l. Cové strain were exposed to the permethrin and deltamethrin test papers. Percentage mortality was 8% and 12% respectively demonstrating a high frequency of pyrethroid resistance.

In the resistance intensity bottle bioassays with technical grade alpha-cypermethrin the LC50 were 0.0004μg for Kisumu and 0.083μg for Cové strain demonstrating a resistance ratio of 207 (CI: 120–316) for *An gambiae* s.l. Cové (Table 1).

**Table 1:** Intensity of pyrethroid (alpha-cypermethrin) resistance in *Anopheles gambiae* s.l. from Cové. Lethal concentrations are expressed in μg/ml.

Strain	Slope (S.E.)	LC50 (95% CI)	LC95 (95% CI)	Resistance ratio at LC50 (95% CI)
Kisumu	1.08 (0.15)	0.0004 (0.000–0.017)	0.011 (0.002–3.644)	-
Cové	1.32 (0.35)	0.083 (0.048–0.106)	1.493 (0.881–4.423)	207 (120–316)

### Experimental hut trial

#### Mosquito entry and exiting from huts

**Fig 1:**
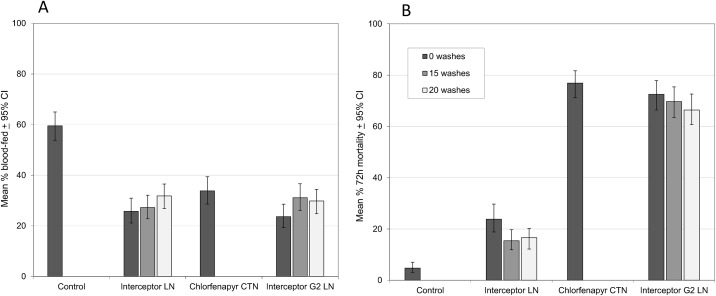
Experimental hut trials against wild *Anopheles gambiae* s.l. with Interceptor^*®*^ G2 and other nets. (A) Percentage blood-feeding, (B) Percentage mortality.

The efficacy of each type of net (Interceptor^®^ G2 LN, Interceptor^®^ LN and chlorfenapyr CTN) was assessed under carefully controlled household-like conditions. Using volunteers sleeping overnight in experimental huts in Cové, the nets were washed up to 20 times and tested for their ability to prevent biting and kill wild, host-seeking pyrethroid-resistant An. gambiae s.l. mosquitoes) that entered the huts to feed [19]. These experimental hut studies (presented in Tables 2 and 3 and Fig 1) enabled the mosquito nets to be evaluated for their capacity to deter mosquito entry, inhibit biting and blood-feeding, and kill the mosquito vector.

**Table 2:** Number of wild *Anopheles gambiae* s.l. females entering and leaving the huts in the experimental hut trial.

Treatment	Number of washes	Total females caught	Average caught per night (geometric mean)	Percentage in net (CI)	Percentage exiting (CI)	Induced exiting %
Untreated net	0	673	9.4 (6.7)^a^	40 (34–46)^a^	32 (27–36)^a^	-
Chlorfenapyr CTN	0	713	10.4 (6.4)^a^	24 (19–29)^d^	50 (45–55)^c^	27
Interceptor LN	0	631	9.3 (5.9)^a^	10 (7–13)^e^	59 (54–64)^bd^	40.4
	15	799	10.4 (7.6)^ac^	22 (18–27)^bd^	51 (46–56)^c^	28.6
	20	950	13.4 (9.9)^bc^	19 (15–24)^bc^	51 (46–56)^c^	28.6
Interceptor G2 LN	0	697	10.4 (6.7)^ad^	10 (17–13)^e^	62 (57–67)^d^	44.7
	15	616	9.1 (6.9)^a^	18 (14–23)^bc^	59 (53–64)^bd^	39.7
	20	929	13.0 (8.5)^bcd^	16 (12–20)^c^	56 (51–60)^bc^	35.4

Values in the same column not sharing a letter superscript differ significantly (P < 0.05)

**Table 3:** Blood feeding inhibition, personal protection and mortality rates of *Anopheles gambiae* s.l. due to insecticide treated nets in the experimental hut trial.

Treatment	Number of washes	Blood-feeding inhibition % (CI)	Personal protection %	Mortality %, corrected for control mortality (CI)	Mortality as % of all mortality
Untreated net	0	0^a^	0^a^	0^a^	56
Chlorfenapyr CTN	0	43 (34–52)^b^	36.7^e^	76 (70–81)^f^	67
Interceptor LN	0	57 (48–65)^c^	62.5^c^	76 (70–81)^f^	67
	15	54 (46–62)^c^	47.1^bce^	11 (8–16)^d^	69
	20	47 (38–54)^b^	22^ab^	13 (9–17)^d^	70
Interceptor G2 LN	0	60 (52–68)^c^	59.2^c^	71 (65–77)^c^	72
	15	48 (39–56)^bc^	53.2^bce^	68 (62–74)^bc^	67
	20	50 (41–58)^bc^	34.4^be^	65 (58–71)^b^	67

Values in the same column not sharing a letter superscript differ significantly (P<0.05)

An average of 858 female mosquitoes per treatment were collected over 72 nights of the trial (Table 2). The majority (98.8%) were *Anopheles coluzzii* Coetzee & Wilkerson (formerly *Anopheles gambiae* M form). There were no major differences in the numbers collected between treatments. Although more were collected from huts with Interceptor^®^ LN and Interceptor G2 LN that had been washed 20 times, there was no evidence of deterred hut entry with treated nets compared to the untreated net control (Table 2).

Up to 40% of mosquitoes were collected from inside the nets (6 holes had been deliberately cut into the sides of each net to mimic wear and tear). The greatest number were found inside the untreated nets and the fewest inside the unwashed Interceptor^®^ LN and Interceptor^®^ G2 LN presumably due to excito-repellent effect of the pyrethroid.

Exiting rates into the veranda trap were generally higher with the pyrethroid treated nets (Interceptor^®^ LN and Interceptor^®^ G2 LN) and least with the untreated net. There was an inverse trend of reduced exiting with increasing number of washes. Chlorfenapyr also appeared to stimulate exiting compared to the untreated net.

#### Blood feeding inhibition and personal protection

Percentage blood feeding of mosquitoes was 47–60% less with Interceptor^®^ G2 LN and Interceptor^®^ LN relative to the untreated net (Fig 1a). Blood feeding inhibition in mosquitoes and personal protection from mosquito bites were both significant (P = 0.0001) across all treatments relative to the untreated net (Table 3). There was no significant difference in percentage blood feeding between Interceptor^®^ G2 LN and Interceptor^®^ LN over the 20 washes (P = 0.26).

There was an inverse trend of reduced personal protection with increasing number of washes that was significant for both Interceptor^®^ G2 LN (P = 0.012) and Interceptor^®^ LN (P = 0.0001). The chlorfenapyr CTN provided 37% more personal protection than the untreated net (P = 0.0001). There was no difference in personal protection between Interceptor^®^ LN and Interceptor^®^ G2 LN at any wash stage (P = 0.46).

#### Mosquito mortality

Mortality with the untreated control net after a 72h holding period was 5% (Fig 1b). The mortality induced by the alpha-cypermethrin LN (Interceptor LN) against pyrethroid resistant *An gambiae* s.l was only 24% when unwashed (20% after correcting for control mortality) decreasing to 17% after 20 washes (13% after correction) (Table 3). Mortality was highest with the chlorfenapyr CTN (77%). Mortality with the unwashed chlorfenapyr-alpha-cypermethrin LN (Interceptor G2 LN) was 73% (71% after correction). There was no significant change in mortality with Interceptor^®^ G2 LN over 15 washes (recorded as 70% or 68% after correction) (P = 0.31) but a small change was evident after 20 washes (66%, or 65% after correction) which was significant (P = 0.02). This indicates that the chlorfenapyr component made the largest contribution to mortality of pyrethroid resistant *An gambiae* s.l. and the formulation was wash resistant over 20 washes. Of all mosquitoes killed by the LN/CTN treatments, 67 to 72% were collected dead from the huts on the morning of collection. This proportion did not differ between alpha-cypermethrin-only and chlorfenapyr-only treatments (Table 3). The insecticide-induced mortality was more ‘immediate’ (within 6-12h of contact with the net) than ‘delayed’ (after 24-72h holding) and the delayed mortality was not chlorfenapyr-specific but applied to pyrethroid too.

### Chemical analysis and bio-activity of nets tested in experimental huts

**Fig 2:**
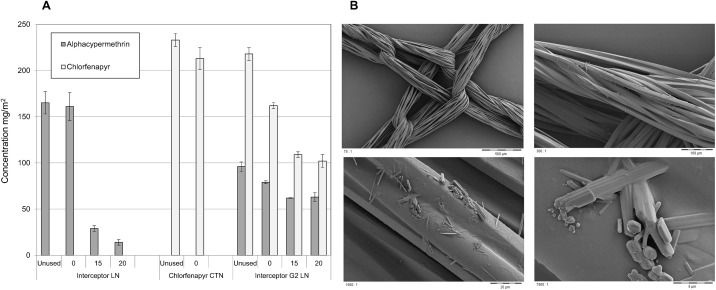
Insecticide content of Interceptor^*®*^ G2 LN and other nets washed up to 20 times used in the hut trial. (A) Chemical analysis (mean ± standard error). (B) Scanning electron microscope images of Interceptor^®^ G2 (courtesy of BASF SE) showing chlorfenapyr (elongate) and alphacypermethrin (short) crystals on netting fibres.

The insecticide content of representative nets before washing, after washing and after trialing in the huts are shown in Fig 2a. Chemical analysis confirms that the alpha-cypermethrin content of unused Interceptor^®^ LN (165 ± 12 mg/m^2^) and unused Interceptor^®^ G2 LN (96 ± 5 mg/m^2^) were within ±25% of target specification and hence met the tolerance limits required by WHO [19]. The initial chlorfenapyr content of the CTN (233 ± 7 mg/m^2^) and Interceptor G2 LN (218 ± 7 mg/m^2^) were also within ± 25% of specification. Nets used in the experimental huts could only be sampled for chemical analysis after trial completion. Among these, the alpha-cypermethrin content of the unwashed Interceptor^®^ LN (161 ± 15 mg/m^2^) and unwashed Interceptor^®^ G2 LN (79 ± 1.5 mg/m^2^) and the chlorfenapyr content of the CTN (213 ± 12 mg/m^2^) and unwashed Interceptor^®^ G2 LN (162 ± 3 mg/m^2^) were within target specification. After 15 and 20 washes the alpha-cypermethrin content of Interceptor^®^ LN had decreased to 29 and 14 mg/m^2^ respectively or 18% and 9% of the initial content. After 15 to 20 washes of Interceptor^®^ G2 LN the alpha-cypermethrin content had decreased to 63 ± 5 mg/m^2^ or 80% of the initial content of unwashed Interceptor^®^ G2 LN, indicating a stronger binding of pyrethroid in Interceptor^®^ G2 LN compared to Interceptor^®^ LN. After 15 to 20 washes the chlorfenapyr content of Interceptor^®^ G2 LN had decreased to 102 ± 7 mg/m^2^ or 63% of the unwashed net, indicating a high wash retention of chlorfenapyr on polyester netting.

The small decrease in percentage mortality over 20 washes with Interceptor^®^ LN (from 20 to 13%) appears to be independent of the change in alpha-cypermethrin content (from 161 to 14 mg/m^2^) which decreased by an order of magnitude over this wash interval. This reflects how pyrethroid resistance enabled mosquitoes to survive across a wide range of insecticide concentrations. In contrast, the 63% reduction of chlorfenapyr content on Interceptor^®^ G2 LN after 15–20 washes (from 162 to 102 mg/m^2^) led to only a small change in mortality (from 71 to 65%) indicating the minimum effective content of chlorfenapyr was below this range.

Fig 2b shows the Interceptor^®^ G2 LN netting under the scanning electron microscope with pictures taken at different magnifications. Individual crystals of active ingredient are visible on the surface, where they are accessible to mosquito contact on touching the netting. Some of the binder formulation is perceived to bridge individual fibers of the multifilament yarn used for producing polyester netting.

### Supplementary laboratory bioassays

#### Cone and tunnel tests

The purpose of the supplementary bioassays was to sample netting from the Interceptor^®^ G2 LN, Interceptor^®^ LN and chlorfenapyr CTN that had been used in the experimental hut trial to: 1) evaluate efficacy against pyrethroid resistant and susceptible strains in mosquito bioassay, and 2) examine the capacity of laboratory bioassay to predict the performance of Interceptor^®^ G2 LN and its constituent insecticides under experimental hut conditions.

**Fig 3:**
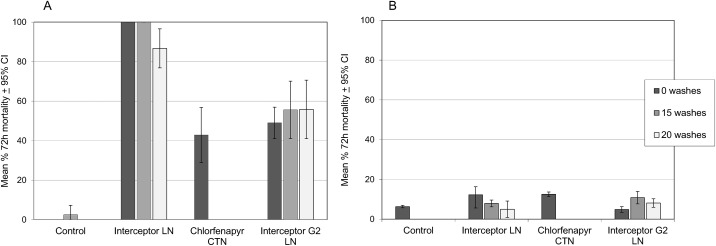
Mortality rates of *Anopheles gambiae* in cone tests with 3 min exposure to Interceptor^*®*^ G2 and other nets. (A) Susceptible Kisumu strain. (B) Resistant Cové strain.

Fig 3a and 3b present the proportions of pyrethroid-susceptible and resistant mosquitoes that were killed 72 h after a 3 minute exposure to insecticide treated netting in WHO cone bioassays. On testing unwashed netting against the susceptible Kisumu strain, mortality was 100% with Interceptor^®^ LN, 58% with Interceptor^®^ G2 LN and 42% with chlorfenapyr CTN. On testing unwashed netting against the pyrethroid resistant Cové strain, mortality did not exceed 12% with any of Interceptor^®^ LN and G2 LN or CTN. Washing Interceptor^®^ LN and Interceptor^®^ G2 20 times made little difference to the proportions killed of either mosquito strain relative to unwashed LN.

**Fig 4:**
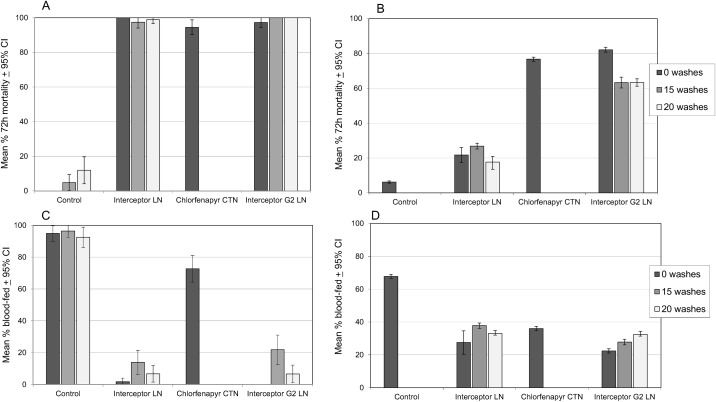
Response of *Anopheles gambiae* in tunnel tests with Interceptor^*®*^ G2 and other nets. (A) Mortality of susceptible strain. (B) Mortality of resistant strain. (C) Blood-feeding rate of susceptible strain. (D) Blood-feeding rate of resistant strain.

Fig 4a and 4b show the proportions of pyrethroid-susceptible and resistant *An*. *gambiae* killed 72h after exposure to the same pieces of insecticide treated netting in overnight tunnel tests. Tests against the pyrethroid susceptible Kisumu strain recorded high mortality with each type of netting (94–100%). Tests against the pyrethroid resistant Cové strain recorded 22% mortality with the unwashed Interceptor^®^ LN, 82% with the unwashed Interceptor^®^ G2 and 77% with the chlorfenapyr CTN. Tests with LN washed up to 20 times showed up to 25% reduction in mortality.

Blood feeding inhibition in the tunnel tests with the susceptible Kisumu strain ranged from 77–100% and was high for both types of LN (Fig 4c). Blood feeding inhibition with the resistant strain was less, ranging from 44–67% and was similar for both types of LN (Fig 4d). The feeding inhibition seemed largely due to the pyrethroid although chlorfenapyr was also inhibitory to some extent in the CTN.

Comparing cone and tunnel test mortality, resistance to pyrethroid in the Cové strain was strongly expressed in each type of bioassay system. The efficacy of the chlorfenapyr CTN was strongly expressed in the tunnel test but was poorly expressed in the cone test; this trend was consistent in both the susceptible and resistant strain and was therefore independent of pyrethroid resistance. The efficacy of Interceptor G2, being a mixture of chlorfenapyr and pyrethroid, was more evident in the tunnel than the cone and higher in the pyrethroid susceptible than the resistant strain.

#### Comparison with hut trial results

Comparing the laboratory bioassay results on the pyrethroid resistant strain with the experimental hut results on the pyrethroid resistant wild population, both types of bioassay predicted the response in the hut to the pyrethroid-only LN, the tunnel more than the cone. Mortality was 12% in the cone, 22% in the tunnel and 24% in the hut against the unwashed Interceptor LN and 5% in the cone, 18% in the tunnel and 17% in the hut against the 20 times washed Interceptor LN. However, when tested against the chlorfenapyr CTN or mixture LN, the tunnel test was the better predictor of hut mortality than was the cone. Mortality with the unwashed chlorfenapyr CTN was 12% in the cone, 77% in the tunnel and 77% in the hut, and with unwashed Interceptor G2 it was 5% in the cone, 82% in the tunnel and 72% in the hut; the trend between assay systems was similar with the 20 times washed Interceptor^®^ G2.

The blood feeding rates and feeding inhibition with the resistant Cové strain in the tunnel (Fig 4d) were similar to the blood feeding rates and feeding inhibition observed with wild Cové *An gambiae* in the experimental huts (Fig 1a).

This series of tests demonstrates that the chlorfenapyr component of Interceptor^®^ G2 LN makes the major contribution to controlling pyrethroid resistant *An gambiae*. The tunnel tests were highly predictive of efficacy in experimental huts whilst cone bioassays were poorly predictive. The tunnel tests against resistant and susceptible strains showed that Interceptor^®^ G2 LN goes a long way to restoring the efficacy of LN against pyrethroid resistant mosquitoes.

## Discussion

Earlier experimental hut trials of mosquito nets hand treated with alpha-cypermethrin and chlorfenapyr formulations in aqueous solution provided the proof of concept that mixtures of the two insecticides on nets would kill and protect against African mosquitoes in regions where the development of pyrethroid resistance is undermining pyrethroid treated nets and threatening malaria control [12, 13]. Hand treated nets are impractical at scale and have been superseded by long lasting insecticidal nets that can withstand multiple washes and stay effective for the lifespan of the net [1, 19]. The objective of the present study was to evaluate in small-scale field trial—with supporting exploratory bioassay techniques—the efficacy and wash resistance of the long lasting mixture net, Interceptor G2 LN, compared to standard pyrethroid-only LN and to determine whether the mixture LN meets the efficacy criteria set by the World Health Organization for malaria control purposes [19, 20].

Experimental hut trial is the gold standard technique for evaluating the efficacy of LN under controlled household conditions. In our trial the mortality of host-seeking *An*. *gambiae* s.l. exposed to the standard pyrethroid-only Interceptor^®^ LN was less than 20%. This low mortality response is typical of other pyrethroid-only LN evaluated in Benin and is because the main vector species *An*. *coluzzii* has developed high-level resistance to alpha-cypermethrin (207 fold) through a combination of L1014F *kdr* and CYP6P3 P450 mechanisms [8, 9]. Such was the strength of the resistance that a 10-fold difference in the surface content of alpha-cypermethrin between the unwashed and 20-times washed Interceptor^®^ LN made little or no difference to the poor efficacy of the net or to the high proportion of mosquitoes surviving (82% and 89% over 20 washes). To put this result into context, comparison can be made with an earlier hut trial in an area of pyrethroid susceptibility (located in Tanzania) against the same unwashed and 20 times washed Interceptor^®^ LN treatments when only 8% and 24% of host-seeking *An*. *gambiae*, respectively, survived exposure [24].

Interceptor^®^ G2 LN restored the capacity of long lasting insecticidal nets to control highly resistant populations of *An*. *gambiae* s.l. The demonstration of 71% mortality with the unwashed mixture LN, decreasing over 20 washes by only 6% approaches the high level of efficacy once shown by pyrethroid-only LN when *An*. *gambiae* populations were fully susceptible [24]. Chemical analysis and scanning electron micrographs confirmed that both insecticides were closely bound to netting fibres by the formulation and were wash resistant and bio-available. To our knowledge this is the first demonstration of a non-pyrethroid insecticide to be rendered wash resistant through binding technology within a LN ‘coating’ formulation and is a significant achievement in its own right. Chlorfenapyr made the larger contribution to mosquito mortality in the hut trial, whilst the alphacypermethrin component made an important contribution to blood feeding inhibition and personal protection, as indicated by the similarity of response between mixture LN and pyrethroid-only LN. Blood-feeding inhibition ranged from 60% to 50% with the unwashed and 20-times washed Interceptor G2 LN as compared with 57% to 47% with Interceptor LN. This contrasts with 74% to 66% blood-feeding inhibition once observed with unwashed and 20-times washed Interceptor LN in hut trials in Tanzania which suggests there is some loss of personal protection in Benin due to resistance relative to regions of pyrethroid susceptibility [24]. While Interceptor G2 would continue to provide personal protection to individual users in pyrethroid resistant areas it is recommended that deployment is done to universal coverage so entire villages can benefit from the community protection which arises from the mass killing of mosquito populations when an effective LN is taken to scale and which a standard pyrethroid-only LN would no longer provide. Beyond Benin, deployment of the mixture LN should extend and restore effectiveness in the many places being compromised by resistance mediated by *kdr* in combination with metabolic mechanisms [3–5, 9, 25].

Chlorfenapyr has a reputation for slow action and ‘delayed’ toxic activity 2–3 days post exposure [11, 26]. This characteristic is particularly evident in daytime bioassays such as the cone test but not necessarily observed in field situations. For example, in the present hut trial two-thirds of total mortality had occurred by the morning after exposure. Chlorfenapyr seems to be more toxic to mosquitoes during exposure at night when cellular respiration and metabolic activity, under control of their circadian rhythm, are at their peak [26]. With exposure to chlorfenapyr only occurring when anophelines are actively host seeking, the mosquito net is an ideal means of optimizing exposure to the time when mosquitoes are most vulnerable to this insecticide. With most mosquitoes succumbing within hours of exposure, this insecticide is no less potent than many neurotoxic insecticides.

To meet the WHO Pest Evaluation Scheme criteria for recommendation as a long lasting insecticidal net the candidate net should attain a threshold efficacy in Phase I cone or tunnel test against a standard insecticide susceptible strain [19]. Interceptor G2 LN did not achieve the WHO cone test threshold of 80% mortality but it did meet the tunnel test threshold of 80% mortality and 90% blooding feeding inhibition in LN washed 20 times, and would therefore pass Phase I. The cone test was a poor predictor of Interceptor G2 and chlorfenapyr CTN efficacy in experimental huts. The tunnel test was a better predictor because exposure occurs at night when host seeking mosquitoes are more vulnerable to chlorfenapyr. This raises a question about the suitability of daytime bioassay techniques like the cone test as a screening tool for identifying toxic activity of insecticides which are non-neurotoxic. Novel, effective insecticides such as the pyrroles might easily be missed during initial screens. The tunnel test which mimics insecticide exposure as it occurs in the field at night against host seeking mosquitoes is a more reliable technique regardless of the mode of action of the insecticide under test and should therefore be raised in importance and used more widely.

To attain the bioassay efficacy criteria set by the WHO Vector Control Action Group for recommendation against pyrethroid resistant mosquitoes the mixture LN is expected to attain 25% improvement in mortality or inhibition of blood feeding after 20 standardized washes relative to the pyrethroid-only LN [20]. Interceptor G2 failed to meet this threshold in cone test, for the reasons already discussed, but attained a 3-fold improvement in mortality over the pyrethroid-only LN in the tunnel tests which is well above the VCAG threshold.

The WHO cone and tunnel tests conducted on nets previously used in the experimental hut trials help to interpret hut trial results against wild free-flying mosquitoes. In the present Phase II experimental hut trials Interceptor G2 LN attained: 1) the WHOPES efficacy criteria of mortality and blood feeding inhibition equal or better than standard pyrethroid-only LN [19], and 2) the WHO VCAG efficacy criteria of mortality or blood feeding inhibition significantly better than pyrethroid-only LN against local highly resistant mosquitoes [20]. Relative to standard pyrethroid-only Interceptor LN, the Interceptor G2 LN demonstrated a 3–4 fold improvement in percentage mortality. This represents a major advance over the pyrethroid-only LN that are currently being deployed by malaria control programs.

Apart from standard pyrethroid-only LN the only long-lasting net to be currently recommended by WHOPES is co-impregnated with the synergist PBO (piperonyl butoxide). This synergist is able to neutralize certain kinds of MFO responsible for metabolic resistance. In hut trials in Benin, the PBO LN, PermaNet 3.0 killed 44% *A*. *gambiae* (control corrected) when unwashed but after 20 washes killed only 18% which was a similar proportion killed by the 20 times washed pyrethroid-only LN [8]. This contrasts with the 70% killed (control corrected) by the unwashed Interceptor^®^ G2 LN in the present trial and 64% killed after 20 washes.

On the basis of these findings Interceptor^®^ G2 should attain WHOPES interim approval as a LN for use in malaria control. Both types of LNs trialed in the experimental huts showed little or no decrease in chemical content by the end of trial. Full WHOPES approval would require Phase III trials of effectiveness over 3 years of household use. Previous Phase III studies of Interceptor LN and comparison of outcomes of experimental hut and household trials showed that alpha-cypermethrin losses after 3 years of household use were consistent with alpha-cypermethrin losses after 20 Phase II standardized washes and trialing in experiment huts [27]. If this relationship holds true for Interceptor^®^ G2 too, the satisfactory Phase II outcome of the present experimental hut trial would predict a similar outcome following 3 years of household use.

## Conclusion

Selection of resistance to pyrethroid insecticides is the gravest threat to future progress in malaria control. The development of new insecticides suitable for long lasting nets is vital for preservation of this incomparable vector control tool. The mixture LN Interceptor^®^ G2 surpassed the efficacy and wash-resistance thresholds set by WHO and demonstrated capacity to protect individual users and control highly pyrethroid-resistant mosquitoes bearing *kdr* and metabolic mechanisms in small-scale field trial. Roll-out of Interceptor^®^ G2 is urgently required to meet the needs of malaria control, particularly in areas affected by increasing levels of insecticide resistance.

## Supporting Information

S1 Table Experimental hut data, excel file. (XLSX)

## References

[1] World Health Organisation (2015) World Malaria Report 2015. Geneva: WHO http://www.who.int/malaria/publications/world-malaria-report-2015/report/en/

[2] World Health Organisation (2012) Global Plan for Insecticide Resistance Management. Geneva: WHO http://whqlibdoc.who.int/publications/2012/9789241564472_eng.pdf

[3] Knox TB, Juma EO, Ochomo EO, Pates Jamet H, Ndungo L, Chege P, et al (2014) An online tool for mapping insecticide resistance in major Anopheles vectors of human malaria parasites and review of resistance status for the Afrotropical region. Parasit Vectors 7: 76 10.1186/1756-3305-7-76 24559061 PMC3942210

[4] Wondji CS, Coleman M, Kleinschmidt I, Mzilahowa T, Irving H, Ndula M, et al (2012) Impact of pyrethroid resistance on operational malaria control in Malawi. Proc Natl Acad Sci U S A 109: 19063–70. 10.1073/pnas.1217229109 23118337 PMC3511128

[5] N'Guessan R, Corbel V, Akogbéto M, Rowland M (2007a) Reduced efficacy of insecticide-treated nets and indoor residual spraying for malaria control in pyrethroid resistance area, Benin. Emerg Infect Dis 13: 199–206. 17479880 10.3201/eid1302.060631 PMC2725864

[6] Asidi A, N'Guessan R, Akogbéto M, Curtis C, Rowland M. (2012) Loss of household protection from use of insecticide-treated nets against pyrethroid-resistant mosquitoes, Benin. Emerg Infect Dis 18: 1101–6. 10.3201/eid1807.120218 22709930 PMC3376816

[7] West PA, Protopopoff N, Wright A, Kivaju Z, Tigererwa R, Mosha FW, et al (2015) Enhanced protection against malaria by indoor residual spraying in addition to insecticide treated nets: is it dependent on transmission intensity or net usage? PLoS One 10: e0115661 10.1371/journal.pone.0115661 25811379 PMC4374910

[8] N'Guessan R, Asidi A, Boko P, Odjo A, Akogbeto M, Pigeon O, et al (2010) An experimental hut evaluation of PermaNet 3.0, a deltamethrin-piperonyl butoxide combination net, against pyrethroid-resistant Anopheles gambiae and Culex quinquefasciatus mosquitoes in southern Benin. Trans R Soc Trop Med Hyg 104: 758–65. 10.1016/j.trstmh.2010.08.008 20956008

[9] Ngufor C, N'Guessan R, Fagbohoun J, Subramaniam K, Odjo A, Fongnikin A, et al (2015) Insecticide resistance profile of Anopheles gambiae from a phase II field station in Cové, southern Benin: implications for the evaluation of novel vector control products. Malar J 14: 464 10.1186/s12936-015-0981-z 26581678 PMC4652434

[10] Black BC, Hollingsworth RM, Ahammadsahib KI, Kukel CD, Donovan S (1994) Insecticidal action and mitochondrial uncoupling activity of AC-303,630 and related halogenated pyrroles. Pestic. Biochem. Physiol 50: 115–128.

[11] N’Guessan R, Boko P, Odjo A, Akogbeto M, Yates A, Rowland M. (2007b) Chlorfenapyr: a pyrrole insecticide for the control of pyrethroid or DDT resistant Anopheles gambiae (Diptera: Culicidae) mosquitoes. Acta Trop 102: 69–78. 17466253 10.1016/j.actatropica.2007.03.003

[12] Oxborough RM, Kitau J, Matowo J, Feston E, Mndeme R, Mosha FW, et al (2013) ITN mixtures of chlorfenapyr (pyrrole) and alphacypermethrin (pyrethroid) for control of pyrethroid resistant Anopheles arabiensis and Culex quinquefasciatus. PLoS One 8: e55781 10.1371/journal.pone.0055781 23409042 PMC3567122

[13] N'Guessan R, Ngufor C, Kudom A, Boko P, Odjo A, Rowland M. (2014) Mosquito nets treated with a mixture of chlorfenapyr and alphacypermethrin control pyrethroid resistant Anopheles gambiae and Culex quinquefasciatus mosquitoes in West Africa. PLoS One 9: e87710 10.1371/journal.pone.0087710 24498360 PMC3912058

[14] Curtis CF (1985) Theoretical models of the use of insecticide mixtures for the management of resistance. Bull Ent Res 75: 259–265.

[15] Mosha F, Lyimo I, Oxborough R, Malima R, Temu F, Matowo J, et al (2008) Experimental hut evaluation of the pyrrole insecticide chlorfenapyr on bed nets for the control of Anopheles arabiensis and Culex quinquefasciatus. Trop Med Int Health 13: 644–652. 10.1111/j.1365-3156.2008.02058.x 18419583

[16] Oliver SV, Kaiser ML, Wood OR, Coetzee M, Rowland M, Brooke BD. (2010) Evaluation of the pyrrole insecticide chlorfenapyr against pyrethroid resistant and susceptible Anopheles funestus (Diptera: Culicidae). Trop Med Int Health 15: 127–31. 10.1111/j.1365-3156.2009.02416.x 19891759

[17] Raghavendra K, Barik TK, Sharma P, Bhatt RM, Srivastava HC, Sreehari U, et al (2011) Chlorfenapyr: a new insecticide with novel mode of action can control pyrethroid resistant malaria vectors. Malar J 10: 16 10.1186/1475-2875-10-16 21266037 PMC3039634

[18] Yuan JZ, Li QF, Huang JB, Gao JF (2015) Effect of chlorfenapyr on cypermethrin-resistant Culex pipiens pallens Coq mosquitoes. Acta Trop 143: 13–7. 10.1016/j.actatropica.2014.12.002 25497774

[19] World Health Organisation (2013) Guidelines for laboratory and field testing of long-lasting insecticidal mosquito nets WHO/HTM/NTD/WHOPES/2013.11. Geneva: WHO.

[20] World Health Organisation (2014) Third meeting of the Vector Control Advisory Group; Geneva, Switzerland 12–14 November 2014. WHO/HTM/NTD/VEM/2015.1. Geneva: WHO.

[21] World Health Organisation (2006) Guidelines for testing mosquito adulticides for indoor residual spraying (IRS) and for treatment of mosquito nets (ITNs). WHO/CDS/WHOPES/GCDPP/2006. Geneva: WHO.

[22] Bagi J, Grisales N, Corkill R, Morgan JC, N'Falé S, Brogdon WG, et al (2015) When a discriminating dose assay is not enough: measuring the intensity of insecticide resistance in malaria vectors. Malar J 14: 210 10.1186/s12936-015-0721-4 25985896 PMC4455279

[23] World Health Organisation (2007) Report of the Tenth WHOPES Working Group Meeting. Review of Spinosad 0.5% GR & 12% SC; Lambda-cyhalothrin 10% CS; K-O Tab 1-2-3; Interceptor™. WHO/CDS/NTD/WHOPES/2007.1. Geneva: WHO.

[24] Malima R, Tungu PK, Mwingira V, Maxwell C, Magesa SM, Kaur H, et al (2013) Evaluation of the long-lasting insecticidal net Interceptor LN: laboratory and experimental hut studies against anopheline and culicine mosquitoes in northeastern Tanzania. Parasit Vectors 6: 296 10.1186/1756-3305-6-296 24499488 PMC4028879

[25] David JP, Ismail HM, Chandor-Proust A, Paine MJ (2013) Role of cytochrome P450s in insecticide resistance: impact on the control of mosquito-borne diseases and use of insecticides on Earth. Philos Trans R Soc Lond B Biol Sci 368: 20120429 10.1098/rstb.2012.0429 23297352 PMC3538419

[26] Oxborough RM, N’Guessan R, Jones R, Kitau J, Ngufor C, Malone D, et al (2015) The activity of the pyrrole insecticide chlorfenapyr in mosquito bioassay: towards a more rational testing and screening of non-neurotoxic insecticides for malaria vector control. Malaria Journal 14: 124 10.1186/s12936-015-0639-x 25879231 PMC4390098

[27] Tungu P, Kirby M, Malima R, Kisinza W, Magesa S, Maxwell C, et al (2016) Interceptor^®^ long-lasting insecticidal net: phase III evaluation over three years of household use and calibration with Phase II experimental hut outcomes. Parasit Vectors 9: 204 10.1186/s13071-016-1490-9 27075874 PMC4831182

